# Src-family kinases in the development and therapy of Philadelphia chromosome-positive chronic myeloid leukemia and acute lymphoblastic leukemia

**DOI:** 10.1080/10428190701713689

**Published:** 2008-01-17

**Authors:** Shaoguang Li

**Affiliations:** The Jackson Laboratory, Bar Harbor, ME, USA

**Keywords:** Src, leukemia, BCR-ABL, dasatinib, imatinib resistant

## Abstract

The BCR-ABL kinase inhibitor imatinib has shown significant efficacy in chronic myeloid leukemia (CML) and is the standard front-line therapy for patients in chronic phase. However, a substantial number of patients are either primarily refractory or acquire resistance to imatinib. While a number of mechanisms are known to confer resistance to imatinib, increasing evidence has demonstrated a role for BCR-ABL–independent pathways. The Src-family kinases (SFKs) are one such pathway and have been implicated in imatinib resistance. Additionally, these kinases are key to the progression of CML and Philadelphia chromosome-positive acute lymphoblastic leukemia (Ph+ ALL). The dual SFK/BCR-ABL inhibitor dasatinib is now clinically available and has markedly greater potency compared with imatinib against native BCR-ABL and the majority of imatinib resistant BCR-ABL mutants. Therefore, this agent, as well as other dual SFK/BCR-ABL inhibitors under development, could provide added therapeutic advantages by overcoming both BCR-ABL– dependent (i.e., BCR-ABL mutations) and – independent forms of imatinib resistance and delaying transition to advanced phase disease. In this review, we discuss the preclinical and clinical evidence demonstrating the involvement of SFKs in imatinib resistance and the progression of CML and Ph+ ALL, as well as the potential role of dual SFK/BCR-ABL inhibition in the management of these diseases.

## Introduction

The constitutively active BCR-ABL tyrosine kinase is the defining molecular abnormality in Philadelphia chromosome-positive (Ph+) chronic myeloid leukemia (CML) and acute lymphoblastic leukemia (ALL) [1–6]. The pathogenic role of BCR-ABL in CML and Ph+ ALL provided the rationale for therapeutic targeting of this signaling protein. Imatinib was the first available BCR-ABL targeted therapy and is currently the standard front-line therapy for CML in chronic phase (CP). However, despite the significant efficacy of this agent, a substantial number of patients are either primarily resistant to treatment or acquire resistance during the course of treatment [7–14]. Additionally, imatinib does not completely eradicate residual leukemic stem cells and progenitors [15,16], which present a persistent risk of disease relapse.

The Src-family kinases (SFKs) have been implicated in BCR-ABL signaling [17,18] and in the progression of CML and Ph+ ALL [19–27]. Furthermore, increasing evidence suggests that SFKs are involved in BCR-ABL-independent forms of imatinib resistance [26,27]. Here we will review the preclinical and clinical evidence demonstrating SFK involvement in BCR-ABL signaling, the transforming activity of BCR-ABL, progression of CML and Ph+ ALL, and imatinib resistance.

## Oncogenic signaling pathways in CML and PH+ ALL

BCR-ABL is a constitutively active, non-receptor tyrosine kinase [2,3,28]. The central role of this oncogenic kinase in the pathogenesis of CML has been well established [3,29]. BCR-ABL initiates numerous signal transduction pathways that influence the growth and survival of hematopoietic cells and collectively induce leukemic transformation, such as STAT5, MEK1/2/ERK1/2, and NF-κB [30]. Several mechanisms have been implicated in the transforming activity of BCR-ABL, including constitutive mitogenic signaling [31] and reduced dependency on external growth factors [32], altered cell adhesion properties [33], and reduced apoptotic potential [34]. Additionally, evidence suggests that BCR-ABL disrupts the DNA repair response [35,36], which may play a role in disease progression by exacerbating genomic instability and promoting the accumulation of additional cytogenetic alterations.

Given the central role of BCR-ABL in the pathogenesis of CML, it is an attractive target for selective kinase inhibition. However, targeting BCR-ABL kinase activity alone may not be sufficient for the management of CML, as downstream pathways of BCR-ABL can be activated independently of BCR-ABL kinase activity [23], thereby leading to imatinib resistance. The SFKs are an example of such a downstream activator, and have been suggested to confer BCR-ABL independence. These non-receptor, intracellular tyrosine kinases regulate signal-transduction pathways involved in cell growth, differentiation, and survival [37–39] and are among the most extensively studied oncogenes in human cancers [40]. There are eight known SFK members (Src, Blk, Fgr, Fyn, Hck, Lck, Lyn, and Yes) with each comprising a unique domain and high-sequence homology in the four Src homology domains (SH1-4) [41]. SFKs exhibit a range of tissue expression patterns and several are primarily expressed in hematopoietic cells (Table I) [39,41].

**TABLE I.** Expression of SFKs in hematopoietic cells [39].

Lineage	SFK member
T cells	Fyn, Lck
B cells	Blk, Fgr, Fyn, Lyn
Myeloid cells	Fgr, Hck, Lyn

SFK, Src-family kinase.

Numerous studies have indicated an association between SFKs and myeloid and lymphoid leukemias [39]. Early research demonstrated the proleukemic potential of SFKs in a variety of hematopoietic cell lines [42–46]. Danhauser-Riedel et al. provided the first data demonstrating that the activity of the SFKs, Lyn and Hck, is increased in hematopoietic cells expressing BCR-ABL [18]. Activation of Hck or other SFK members has been suggested to be required for BCR-ABL-mediated transformation [20,47]. Expression of a kinase defective mutant of Hck blocked BCR-ABL-induced outgrowth of cytokine dependent leukemia cell lines [20]. Furthermore, pharmacologic inhibition of SFKs led to growth arrest and apoptosis in CML cell lines [48]. Recent SFK research has centered on the pathologic role of these signaling molecules in CML and Ph+ ALL, their involvement in disease progression and the development of imatinib resistance.

## Cooperation between BCR-ABL and SFK signaling pathways

**Figure 1.**
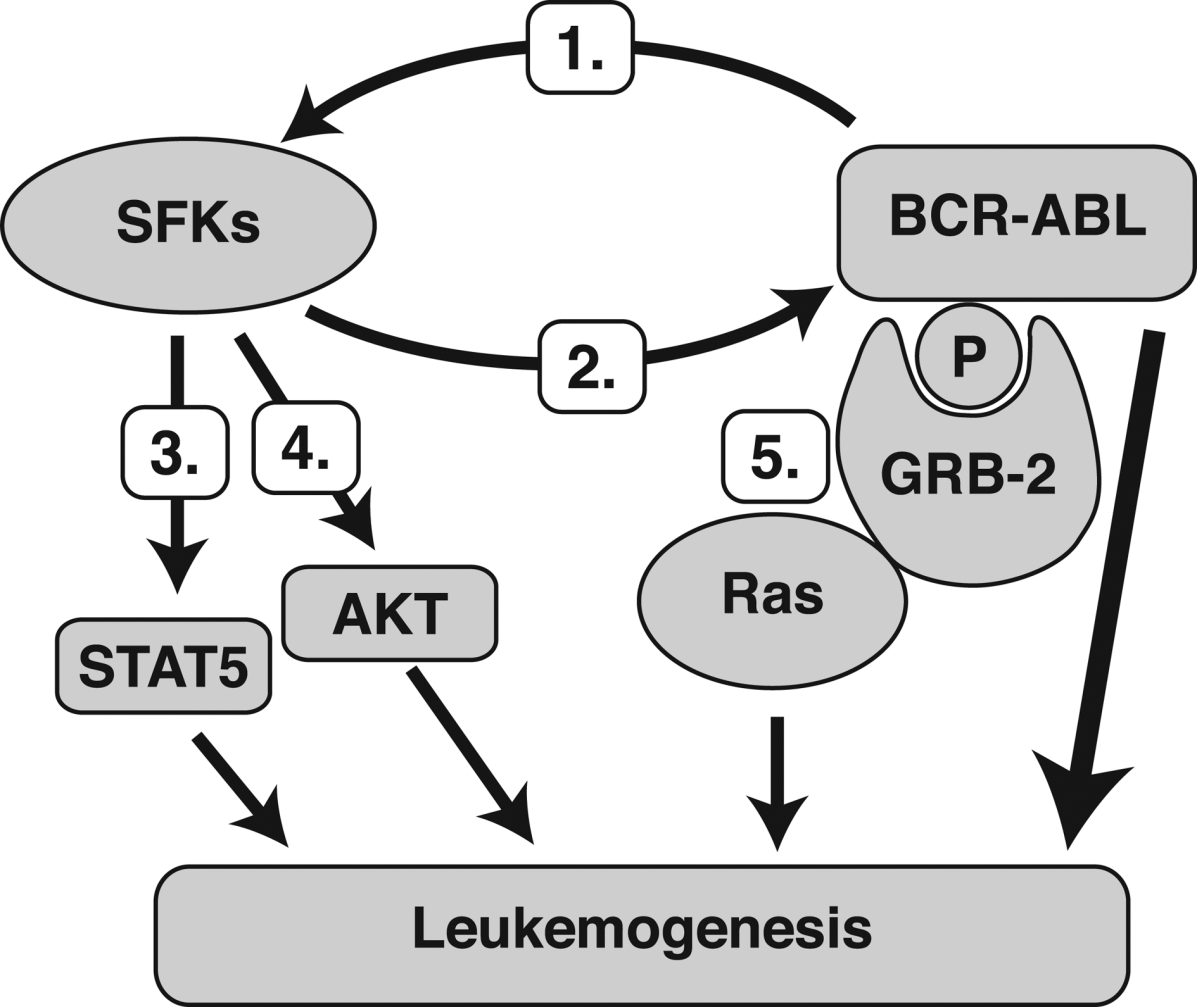
SFKs directly interact with BCR-ABL resulting in (1) activation of SFKs [17,18,25] and (2) augmentation of BCR-ABL kinase activity [49]. Activated SFKs work cooperatively with BCR-ABL in facilitating the growth and progression of leukemia [48,50,51]. Several downstream effectors of SFKs have been proposed to mediate the proleukemic effects, such as (3) STAT5 [50], which is known to activate genes involved in growth factor independence, differentiation, adhesion, and DNA repair [36,52–54] and (4) AKT [55], which is key in regulating cell proliferation and survival in BCR-ABL-dependent cells [56]. (5) Active SFKs also phosphorylate certain tyrosine residues on BCR-ABL to create a binding site for GRB-2. This adaptor protein may link the BCR-ABL pathway to Ras, which is known to activate the MEK/ERK oncogenic signaling cascade [17,48].

SFKs are collaborative oncogenic kinases in BCR-ABL-induced leukemias and may act to couple BCR-ABL to certain downstream signaling pathways involved in leukemic transformation (Figure 1) [17,18,20,47,48]. SFKs are activated through direct interaction with BCR-ABL [17,18,25], and likely involves the release ofintramolecular, auto-inhibitory constraints [17,20,38]. In turn, the activity of BCR-ABL can be enhanced through SFK-mediated phosphorylation, which by Hck of tyrosine residues within the activation loop of ABL was found to increase ABL kinase activity [49]. In another study, it was demonstrated that Hck, Lyn, and Fyn phosphor-ylate multiple tyrosine residues within the SH3-SH2 region of BCR-ABL, and that these phosphorylations are required for full oncogenicity of BCR-ABL in myeloid cell lines. This impact on BCR-ABL function was suggested to occur through the release of an autoregulatory function that holds the kinase domain in an inactive state [47].

## Role of SFKS in the progression of CML to blast crisis

Although BCR-ABL is considered the trigger for malignant transformation in CML, there is evidence of an important role for SFKs in disease progression. Studies from our laboratory demonstrated that the transition of CML to lymphoid blast crisis (LBC) in mice requires the presence of Lyn, Hck, and Fgr [23], and Donato et al. showed that overexpression and/or activation of Hck and Lyn occur during CML progression [21]. Downregulation of Lyn expression by RNAi was found to induce apoptosis in both myeloid and lymphoid blast cells, and this effect was more pronounced in the latter [24]. Although the biology of CML progression is not fully understood and likely involves multiple factors, the studies above clearly implicate SFKs in the development of advanced phase CML, particularly LBC. Furthermore, the involvement of SFKs in disease progression may partially explain the aggressive nature of advanced CML [21] and its relatively poor responsiveness to imatinib [12,57,58].

## Role of SFKS in PH+ ALL

SFKs also appear to play a significant role in the development of Ph+ ALL, which may be independent of BCR-ABL kinase activity. Data from our laboratory with mouse knockout models demonstrated that SFKs are required for induction of BALL by BCR-ABL [25]. We subsequently found that although imatinib had a weak effect on the survival of mice with B-ALL induced by BCR-ABL, coinhibi-tion of BCR-ABL kinase activity and SFKs maintained long-term survival [23]. The independent role of SFKs in B-ALL is further supported by results demonstrating that a SFK inhibitor reduced the viability, and induced apoptosis, in pre-B leukemia cells expressing the imatinib-resistant T315I BCR-ABL mutant [25]. Moreover, while imatinib had no effect in mice with B-ALL induced by T315I BCR-ABL, treatment with the dual Src/ABL inhibitor dasatinib (which is also ineffective against the T315I BCR-ABL mutant) significantly prolonged survival. In addition, this improved survival correlated with SFK inhibition [23]. Collectively, these results indicate that SFKs play a role in B-lymphoid transformation that is not efficiently prevented by inhibiting BCR-ABL kinase activity with imatinib and that dual SFK/ABL inhibition may improve the overall treatment outcome of patients with this disease.

## SFKS and resistance to imatinib

Resistance to imatinib develops rapidly in patients with advanced phase CML and Ph+ ALL [12,57,58]. A number of potential resistance mechanisms have been proposed including BCR-ABL kinase domain mutation, BCR-ABL overexpression, alterations in drug influx and efflux, and as mentioned previously, induction of BCR-ABL-independent pathways [59]. Increasing preclinical and clinical evidence implicates SFKs in imatinib resistance. Upregulation of Lyn and Hck was observed in blasts from patients with imatinib-resistant CML [21]. It was later found that while imatinib effectively reduced activation of BCR-ABL and downstream activation of SFKs in specimens derived from patients with imatinib-sensitive CML, this agent had no effect on SFK activation in samples from resistant patients despite BCR-ABL inhibition. In animal models, the antitumor activity of imatinib was significantly reduced upon loss of imatinib-mediated Lyn inhibition, but concomitant inhibition of SFKs and BCR-ABL recovered this activity [27]. These results further indicated that SFK activity becomes independent of BCR-ABL in progressive disease, thereby resulting in imatinib resistance. Other studies have provided similar evidence of SFK-mediated BCR-ABL independence. The SFKs Lyn and Hck were found to be overexpressed in CML cell lines with BCR-ABL–independent imatinib resistance, and coinhibition of SFKs and BCR-ABL in these cells resulted in an enhanced apoptotic response [21,26]. Dai et al. showed that imatinib-resistant cell lines demonstrated markedly increased expression of Lyn, and treatment with a specific Src inhibitor induced apoptosis in these cells. Furthermore, transfection ofimatinib-sensitive cell lines with a constitutively active form of Lyn conferred resistance to imatinib [22].

SFKs may also mediate imatinib resistance by stabilizing the active form of BCR-ABL as imatinib is unable to bind this conformation [60,61]. As mentioned above, Meyn et al. suggested that SFK-mediated phosphorylations in the SH3-SH2 region of BCR-ABL promote the active conformation through disruption of an intramolecular regulatory component that holds the kinase domain in an inactive state [47]. Consistent with this, Azam et al. found that substitution of one of these SFK-phosphorylated tyrosine residues within the SH3 domain resulted in imatinib resistance [62]. Furthermore, Hck promotes the active conformation through the phosphorylation of specific tyrosine residues within the ABL activation loop, which substantially decreases the sensitivity to imatinib [49,61].

## Dual SFK/BCR-ABL inhibition in CML and PH+ ALL

In CML and Ph+ ALL, treatment based exclusively on the inhibition of BCR-ABL kinase activity (e.g., imatinib and its analogs) will clearly not improve patient outcome if BCR-ABL–independent resistance occurs, and may select for resistant clones [23]. Additionally, imatinib is unable to eradicate BCR-ABL–expressing CD34+ cells [63], which will be necessary to achieve curative therapy. Given the role of SFKs in the development and progression of Ph+ ALL and CML and in BCR-ABL–independent imatinib resistance, dual inhibition of BCR-ABL and SFK will likely prove a more effective treatment strategy. Additionally, this strategy may also suppress the emergence of BCR-ABL–independent clones, delay and possibly prevent transition ofCML to blast crisis (BC), and yield greater activity in advanced CML and Ph+ ALL [23].

As discussed above, preclinical studies have demonstrated that pharmacologic or genetic inhibition of SFKs induces apoptosis and growth arrest in BCR-ABL transformed cells [20,23 – 25,48] and may overcome imatinib resistance [22,40,64]. Moreover, dual inhibitors of BCR-ABL and SFKs may be less susceptible to conformational resistance than imati-nib [65]. Although several such agents are currently in early stage clinical development [66], dasatinib is the most clinically advanced and is the only dual SFK/BCR-ABL inhibitor approved in the United States and Europe for the treatment of patients with imatinib-resistant or -intolerant CML and Ph+ ALL. This novel, orally available, tyrosine kinase inhibitor is structurally unrelated to imatinib, and capable of binding to the ABL kinase domain in multiple conformations [67–69]. This agent has demonstrated 325-fold greater activity against native BCR-ABL in vitro as compared with imatinib, and is active against all imatinib-resistant BCR-ABL mutations with the exception of T315I [67,68]. Additionally, dasatinib also has activity against other oncogenic tyrosine kinases such as c-Kit, platelet-derived growth factor-receptor (PDGFR), and ephrin A-receptor [70–73].

Numerous preclinical studies have indicated that dual SFK/BCR-ABL inhibition with dasatinib is advantageous in CML and Ph+ ALL. As previously mentioned, Donato et al. showed that dasatinib was able to recover the antitumor activity lost with imatinib treatment as a result of BCR-ABL–independent Lyn activation [27]. We recently reported that treatment with dasatinib induces complete remission of Ph+ ALL, and significantly prolongs survival of CML in mice [23]. It was also found that SFKs are required for the progression of CML to LBC, suggesting that treatment with dasatinib could potentially delay the transition of CML from CP to LBC. Additionally, although dasatinib does not kill leukemic stem cells, studies in B-ALL mice suggest that the cytostatic effects of dasatinib on this cell population could prevent leukemic transformation and afford long-term control of the disease [23].

Results from phase I and II trials showed that dasatinib induces rapid and deep responses in imatinib-resistant patients across all phases of CML and Ph+ ALL [74–79]. While it is clear that the more potent activity of dasatinib against native and mutant variants of BCR-ABL is, in part, responsible for its clinical efficacy in imatinib-resistant CML, BCR-ABL–independent effects appear to play a role as well. Dasatinib has shown activity in imatinib-resistant patients with no detectable mutations at baseline [80]. Furthermore, in a randomized clinical trial, dasatinib demonstrated superior efficacy compared to high-dose (HD) imatinib in patients with imatinib-resistant CP CML, and major cytogenetic responses (CyRs) were achieved in 55% (28/51) of patients without a BCR-ABL mutation at baseline compared with 34% (12/35) observed with HD imatinib [79]. A separate study showed that the clinical activity of dasatinib in patients with BCR-ABL –independent imatinib resistance correlated with inhibition of both BCR-ABL and SFKs in primary cell samples taken from the same patients [27]. Dasatinib was also found to be active in patients with CML after failure of imatinib and its analog nilotinib [81]. Notably, hematologic and CyRs were achieved with dasatinib in a substantial number of patients with advanced stage CML and Ph+ ALL [74,76–78]. This latter result is in marked contrast to the limited activity observed with imatinib and nilotinib in LBC CML and Ph+ ALL [66,82], further suggesting a potential role of SFKs in advanced disease. Collectively, the clinical data support the preclinical findings implicating SFKs in CML progression and imatinib resistance, and suggest that dual SFK/BCR-ABL inhibition may be more effective than inhibition of BCR-ABL alone.

## Most appropriate use of Src/ABL inhibitors

Although dasatinib has demonstrated promising results in advanced phase patients, the outcome of treatment in this population is clearly inferior to that observed in CP CML. Additionally, it remains to be determined whether the responses generated in patients with BC CML and Ph+ ALL will be durable. In the phase I trial of dasatinib responses in these patients were described as short-lived [74]; however, improved response durations were reported in phase II studies [77,78]. Moreover, many ofthese patients do not respond to treatment of any kind. This refractory nature is likely due to the accumulation of additional genetic abnormalities that occur with disease progression, conferring multiple and complex mechanisms of resistance which are not fully understood.

This raises the question of when it is most appropriate to begin treatment with dasatinib. The results in our mouse CML model demonstrating that SFKs are required for progression to BC and that dasatinib significantly prolonged survival compared to imati-nib, suggest that early and continuous treatment with dasatinib in patients with CP CML may provide the greatest therapeutic benefit [23]. This strategy could more effectively prevent disease progression as well as the emergence of drug resistance, through suppression of leukemic stem cell transformation. Thus, while imatinib is currently the standard first-line therapy for newly diagnosed patients in CP, dasatinib should also be considered in these patients.

Earlier treatment with dasatinib may also prove beneficial given its greater potency, which could allow for more rapid achievement of the treatment goals, that is, complete CyR and major molecular response (MMR) [83]. Several studies have shown that both overall survival and progression-free survival is improved in patients who achieve a CyR at 3 or 6 months [8,84–88]. Similarly, early molecular responses are also associated with better outcome [89]. Indeed, dasatinib has shown impressive activity as front-line therapy in CP CML. Complete CyR rates at 3, 6, and 12 months were 77%, 92%, and 95%, respectively, and MMRs were achieved in 19% of patients at 6 months which increased to 32% at 12 months [90]. Long-term follow-up from this study and head-to-head studies evaluating dasatinib versus imatinib as first-line treatment are necessary to determine whether earlier treatment with dasatinib will improve the outcome of patients with CP CML.

## Conclusions

Overall, preclinical and clinical evidence has demonstrated an important role for SFKs in the progression of CML and Ph+ ALL and the development of imatinib resistance. Compared with other BCR-ABL inhibitors, such as imatinib and nilotinib, the anti-SFK activity of dasatinib and other dual SFK/BCR-ABL inhibitors could provide added therapeutic advantages by overcoming both BCR-ABL-dependent and – independent imatinib resistance. Furthermore, long-term suppression of leukemic stem cells with dasatinib may reduce the emergence of resistant clones, translating to more durable responses and improved outcome. Long-term follow-up of dasatinib in imatinib-pretreated as well as early-CP patients will further elucidate the clinical benefit of inhibiting SFKs in CML and Ph+ ALL.
